# Supplementary material for: Neither influence nor selection: Examining co-evolution of political orientation and social networks in the NetSense and NetHealth studies
Source: PLoS One. 2020 May 29;15(5):e0233458. doi: 10.1371/journal.pone.0233458 (PMC7259602; doi:10.1371/journal.pone.0233458)
Supplement: S1 Table — (DOCX) [file pone.0233458.s001.docx]

**S1 Table. Summary statistics of gender, racial identification, and religious preference among NetSense and NetHealth study participants who stayed and dropped out study.**

| NetSense | | | | | | |
| --- | --- | --- | --- | --- | --- | --- |
|  | Wave 2 | | Wave 3 | | Wave 4 | |
|  | Stayer | Dropout | Stayer | Dropout | Stayer | Dropout |
| Count | 178 | 18 | 174 | 22 | 151 | 45 |
| Women | 47% | 44% | 46% | 50% | 47% | 44% |
| Racial identification | | | | | | |
| White | 68% | 72% | 68% | 68% | 68% | 69% |
| Latino | 10% | 6% | 10% | 5% | 11% | 7% |
| African American | 7% | 6% | 6% | 9% | 6% | 9% |
| Asian American | 12% | 11% | 12% | 14% | 12% | 13% |
| Other race | 3% | 5% | 4% | 4% | 3% | 2% |
| Religious preference | | | | | | |
| Catholic | 69% | 72% | 70% | 68% | 70% | 67% |
| Protestant | 12% | 17% | 12% | 18% | 11% | 18% |
| Other religion | 2% | 0% | 2% | 0% | 1% | 4% |
| No religion | 17% | 11% | 16% | 14% | 18% | 11% |
| NetHealth | | | | | | |
|  | Wave 2 | | Wave 3 | | Wave 4 | |
|  | Stayer | Dropout | Stayer | Dropout | Stayer | Dropout |
| Count | 440 | 43 | 401 | 82 | 386 | 97 |
| Women | 48% | 44% | 49% | 45% | 49% | 45% |
| Racial identification | | | | | | |
| White | 65% | 63% | 65% | 62% | 65% | 63% |
| Latino | 13% | 14% | 14% | 15% | 14% | 13% |
| African American | 7% | 2% | 7% | 2% | 7% | 3% |
| Asian American | 9% | 12% | 9% | 10% | 9% | 10% |
| Other race | 6% | 9% | 5% | 11% | 5% | 11% |
| Religious preference | | | | | | |
| Catholic | 73% | 77% | 74% | 72% | 74% | 71% |
| Protestant | 11% | 5% | 10% | 10% | 11% | 10% |
| Other religion | 5% | 5% | 5% | 2% | 5% | 4% |
| No religion | 11% | 13% | 11% | 16% | 10% | 15% |
