# Supplementary material for: Neither influence nor selection: Examining co-evolution of political orientation and social networks in the NetSense and NetHealth studies
Source: PLoS One. 2020 May 29;15(5):e0233458. doi: 10.1371/journal.pone.0233458 (PMC7259602; doi:10.1371/journal.pone.0233458)
Supplement: S2 Table — (DOCX) [file pone.0233458.s002.docx]

**S2 Table. The estimated SAO model excluding partisanship in the political orientation equation for the NetHealth study participants.**

|  | NetHealth |
| --- | --- |
|  | Model 2a |
| Political orientation equation | beta  (s.e.) |
| Rate parameter (period 1) | 1.11***  (0.15) |
| Rate parameter (period 2) | 1.18***  (0.19) |
| Rate parameter (period 3) | 0.77***  (0.12) |
| Linear shape | -0.15  (0.33) |
| Quadratic shape | 0.02  (0.09) |
| Woman | -0.13  (0.12) |
| Latino | 0.10  (0.31) |
| African American | 0.06  (0.25) |
| Asian American | 0.14  (0.24) |
| Other race | 0.06  (0.35) |
| Protestant | -0.23  (0.68) |
| Other religion | -0.20  (0.31) |
| No religion | -0.07  (0.35) |
| Parental annual income | 0.02  (0.04) |
| Parental highest degree | 0.00  (0.07) |
| Average similarity  (Peer influence effect) | 3.98  (2.49) |
| In-degree centrality | 0.01  (0.02) |
| Extraversion | -0.01  (0.08) |
| Agreeableness | -0.04  (0.19) |
| Conscientiousness | 0.01  (0.14) |
| Neuroticism | -0.09  (0.14) |
| Openness | -0.07  (0.13) |
| Frequency of discussing politics with friends | -0.04  (0.06) |
| Network equation | beta  (s.e.) |
| Rate parameter (period 1) | 40.70***  (5.13) |
| Rate parameter (period 2) | 36.95***  (7.90) |
| Rate parameter (period 3) | 24.10***  (1.91) |
| Out-degree (density) | -7.09***  (0.77) |
| Reciprocity | 10.24***  (0.95) |
| Transitive triplets | 0.51***  (0.11) |
| Transitive reciprocated triplets | -0.33*  (0.14) |
| Out-degree – activity | -0.48*  (0.22) |
| In-degree – activity | 0.68  (0.58) |
| In-degree – popularity | 0.00  (0.02) |
| In-in degree^(1/2) assortativity | 0.26***  (0.04) |
| Same residence hall | 0.00  (0.16) |
| Woman alter | 0.59**  (0.20) |
| Woman ego | -0.88*  (0.37) |
| Gender homophily selection | 0.17*  (0.07) |
| Same race | 0.13*  (0.05) |
| Same religious preference | 0.04  (0.10) |
| Parental annual income homophily selection | 0.11  (0.11) |
| Parental highest degree homophily selection | 0.05  (0.09) |
| Extraversion homophily selection | 0.09  (0.12) |
| Agreeableness homophily selection | 0.13  (0.43) |
| Conscientiousness homophily selection | 0.13  (0.19) |
| Neuroticism homophily selection | 0.19  (0.21) |
| Openness homophily selection | 0.32*  (0.15) |
| Frequency discussing politics with friends homophily selection | 0.05  (0.08) |
| Same partisanship | -0.06  (0.10) |
| Political orientation alter | 0.11  (0.11) |
| Political orientation ego | -0.12  (0.14) |
| Political orientation homophily selection | 0.28  (0.21) |
| Overall maximum convergence ratio | 0.24 |

† Two-sided *p*<0.1; * Two-sided *p*<0.05; ** Two-sided *p*<0.01; *** Two-sided *p*<0.001
