# Supplementary material for: Neither influence nor selection: Examining co-evolution of political orientation and social networks in the NetSense and NetHealth studies
Source: PLoS One. 2020 May 29;15(5):e0233458. doi: 10.1371/journal.pone.0233458 (PMC7259602; doi:10.1371/journal.pone.0233458)
Supplement: Attachment — Submitted filename: Response to Reviewers.docx [file pone.0233458.s005.docx]

**Response to Reviewers**

PONE-D-19-35811R1

Neither Influence Nor Selection: Examining Co-evolution of Political Orientation and Social Networks in the NetSense and NetHealth Studies

PLOS ONE

We would like to thank the reviewers for their very constructive feedback. Incorporating the reviewer suggestions for revision has resulted in a greatly improved manuscript. Below, we note the concerns of the reviewers and then explain how we responded to each comment. In all cases, reviewer comments are in italics, while our own comments are in plain text.

**Reviewer 2**

*1. 1. The response rates of 100% at the first surveys seem too good to be true; although not impossible. Which universe is used here as the totality? All freshmen?*

––––––––––––––––––––––––––––––––––––––––––––––––––––

The response rate is the number of participants who complete the survey divided by the number of participants in the sample (Babbie 2016). The NetSense study recruited 196 participants and the NetHealth study recruited 483 participants. Therefore, denominators for the response rates are 196 for NetSense study and 483 for NetHealth study.

*2. P. 10 states “A directed tie between person i and person j exists in a given semester if i initiated a communication event (call or text) with j during that semester”. From Table 5, it can be concluded that the average degree dropped from about 4 to about 2 for NetSense and from just over 8 to just over 6 for NetHealth. How is this possible? These numbers do not tally with the description of the network data collection; or else the smartphones used for data collection are only a minor medium of contact for these students.*

––––––––––––––––––––––––––––––––––––––––––––––––––––

The reviewer is right that the average degree dropped over the first two years for both NetSense and NetHealth participants. However, these numbers reflect the real situation. First, when a freshman made the transition from high school to college in an exclusively residential campus like the University of Notre Dame, his or her personal social networks were dramatically restructured and lifelong relationships (both romantic and friendship) began to form. Student personal networks experienced a large amount of turnover during the first semester, and gradually stabilized over time, as indicated by the Jaccard indices shown in Table 5. Second, since each NetSense participant got a free smartphone and phone plan, it is reasonable to expect average degree to drop by about 49% (from 4 to 2) in their fourth semester, compared to NetHealth participants who brought their own smartphones and used their own plans. Third, the average degrees reported in Table 5 are based on social ties between in-study participants, which accounted for 10% to 12% of all communication events via smartphones for NetSense and NetHealth participants. And we found that the number of all communication events via smartphones also decreased over time, the pattern of which proportionally matches that between in-study participants. These numbers are consistent with previous work showing that while people can interact via face-to-face meetings, letters, landlines, smartphones, emails, Internet, and many other ways, the use of one medium for communication between individuals is strongly correlated with communication via other channels (Haythornthwaite, 2005; Onnela et al., 2007).

References:

Babbie, E. 2016. *The Basics of Social Research (7th Edition)*. Boston, MA: Cengage Learning.

Haythornthwaite, C. (2005). Social networks and internet connectivity effects. *Information, Communication & Society, 8(2),* 125–147.

Onnela, J. P., Arbesman, S., Gonzalez, M. C., Barabási, A. L., & Christakis, N. A. (2011). Geographic constraints on social network groups. *PLoS ONE, 6(4),* e16939.
